# A Doubting Thomas

**Published:** 1903-01

**Authors:** L. L. Loggins

**Affiliations:** Willis, Texas


### A Doubting Thomas.

DR. L. L. LOGGINS, WILLIS, TEXAS.

In the December "Red Back", Dr. A. N. Perkins, one of the oldest, most erudite and experienced physicians in the State vehemently pronounces against the mosquito as the chief propagator of malarial poison. I am a native of the mosquito country, and my twenty years' practice has been among swarms of the mosquitoes of this country, and, while it may, in a secondary way, transmit malaria—as the common house fly undoubtedly transmits typhoid fever, I quite agree with the decided opinion expressed by my old friend Dr. Perkins, whom I have known personally for more than thirty years.

Writers are citing the improved conditions with reference to the decrease of yellow fever in Cuba within the last few years; and they invariably point to the discovery of the mosquito as a carrier of the germs, etc., as its antecedent of this joyful consequence. Now, while the accumulated evidence is far stronger against the mosquito with respect to yellow fever than to malarial fever, most of the recent arguments fall flat when we remember that improved sanitary and hygenic conditions drove the yellow fever first from New Orleans and other points long before the mosquito theory was thought of. There may be more in the present mosquito theory than we wot of; it may be the light of a star not yet discovered; but to those of us who have fought and "cussed" mosquitoes all our lives, and have every day treated some of the manifold expressions of malarial poison in dead winter as well as summer it will require far more evidence than is at present before us to convince us that malarial troubles come specially by the mosquito route.

Dr. Perkins refers to the scoffers of Harvey's discovery of the circulation. We all know that Harvey only opened the way for more mature development. I am conservative enough to wait in this instance, but my waiting will be with but little hope.

Dr. R. T. Morris is right—gauze-packing is wrong in theory. It is a foreign body, and all foreign bodies are of course deleterious. If a drainage tube is ever used, certainly it should be withdrawn at the first possible moment of counter-indication. Far more drainage tubes than are necessary are in daily use. Let us get down to nature. Let us use more hot water instead of so many poisonous antiseptics. Dr. F. E. Daniel and compatriots in the medical service of the late civil war did an immense amount of good surgery with no antiseptics. Yea, they had never heard of anything of the kind. If we would gain more lessons from watching a dog lick his sore leg, we would know more of nature and her laws. I believe in clean, aseptic surgery. I have treated too many extremely septic injuries with entire success to believe all the fads that constantly flow from the great centers. I have seen too many "heaps" of amputated limps about army hospitals (1862-1865) and seen too many maimed officers escape with life to believe all these new things. It is true that the germ theory is clearly established, and that success depends on clean surgery, but that a fresh wound can be rendered more efficiently aseptic with any preparation above sterilized hot water, I certainly doubt. Consequently, I would object to any kind of packing.

